# Exploring NLRP3-related phenotypic fingerprints in human macrophages using Cell Painting assay

**DOI:** 10.1016/j.isci.2025.111961

**Published:** 2025-02-05

**Authors:** Matthew Herring, Eva Särndahl, Oleksandr Kotlyar, Nikolai Scherbak, Magnus Engwall, Roger Karlsson, Mikael Ejdebäck, Alexander Persson, Andi Alijagic

**Affiliations:** 1 School of Medical Sciences, Faculty of Medicine and Health, Örebro University, Örebro, Sweden; 2 Inflammatory Response and Infection Susceptibility Centre (iRiSC), Örebro University, Örebro, Sweden; 3 School of Bioscience, Systems Biology Research Centre, University of Skövde, Skövde, Sweden; 4 Man-Technology-Environment Research Center (MTM), Örebro University, Örebro, Sweden; 5 Centre for Applied Autonomous Sensor Systems (AASS), Robot Navigation & Perception Lab (RNP), Örebro University, Örebro, Sweden; 6 Department of Clinical Microbiology, Sahlgrenska University Hospital, Gothenburg, Sweden; 7 Department of Infectious Diseases, Institute of Biomedicine, Sahlgrenska Academy, University of Gothenburg, Gothenburg, Sweden; 8 Nanoxis Consulting AB, Gothenburg, Sweden

**Keywords:** Immunology, Cell biology, Biological sciences research methodologies

## Abstract

Existing research has proven difficult to understand the interplay between upstream signaling events during NLRP3 inflammasome activation. Additionally, events downstream of inflammasome complex formation such as cytokine release and pyroptosis can exhibit variation, further complicating matters. Cell Painting has emerged as a prominent tool for unbiased evaluation of the effect of perturbations on cell morphological phenotypes. Using this technique, phenotypic fingerprints can be generated that reveal connections between phenotypes and possible modes of action. To the best of our knowledge, this was the first study that utilized Cell Painting on human THP-1 macrophages to generate phenotypic fingerprints in response to different endogenous and exogenous NLRP3 inflammasome triggers and to identify phenotypic features specific to NLRP3 inflammasome complex formation. Our results demonstrated that not only can Cell Painting generate morphological fingerprints that are NLRP3 trigger-specific but it can also identify cellular fingerprints associated with NLRP3 inflammasome activation.

## Highlights

• Cell Painting identifies NLRP3-specific phenotypic fingerprints in human macrophages
• LPS priming amplifies phenotypic changes, influencing the response to NLRP3 triggers
• NLRP3 inhibition mitigates phenotypic changes but does not restore baseline
• Each NLRP3 trigger altered a subset of ER-related phenotypic features

## Graphical abstract

**Figure:**
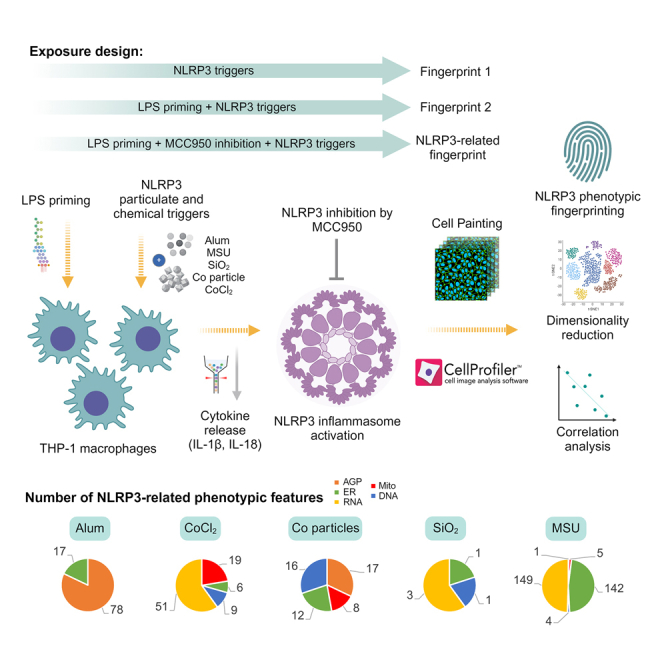

## Introduction

Inflammasome activation is tightly regulated due to its inflammatory nature, generally following a two-step process involving priming and activation. Priming relates to the upregulation of the expression of inflammasome components, like nod-like receptor family pyrin domain containing 3 (NLRP3), pro-caspase-1, and pro-interleukin (IL)-1β, whereas activation pertains to assembly of the inflammasome complex, subsequent cleavage, and activation of pro-caspase-1. Activation further leads to release of mature IL-1β and IL-18 as well as cleavage of gasdermin-D (GSDMD) that facilitates the formation of plasma membrane pores for the release of activated cytokines and triggers pyroptosis, a proinflammatory mode of cell death.^1^^,^^2^ Additionally, priming induces post-translational modification of NLRP3, stabilizing it in an inactive but signal-competent state.^3^ Traditionally, priming was considered to be induced by pathogen-associated molecular patterns (PAMPs), like lipopolysaccharide (LPS), while activation was thought to be initiated by damage-associated molecular patterns (DAMPs), for instance ATP. It has, however, become apparent that various DAMPs, such as TNF and IL-1β, can also function as priming signals and that some PAMPs, like viral RNA, can cause activation of the inflammasome. The most common priming signal used in *in vitro* settings is, however, still LPS.^4^^,^^5^ Additionally, compounds that do not fit into the categories of either PAMP or DAMP can act as potent activators of the NLRP3 inflammasome, including crystals and particulate matter. Exposure to priming and activating signals initiates multiple signaling events upstream of inflammasome complex formation, such as potassium ion (K^+^) or chloride ion (Cl^−^) efflux, calcium ion (Ca^2+^) flux, lysosomal disruption, mitochondrial dysfunction, metabolic changes, and *trans*-Golgi disassembly.^6^^,^^7^^,^^8^^,^^9^^,^^10^^,^^11^^,^^12^^,^^13^^,^^14^ These signals exhibit interconnectedness and overlap, resulting in conflicting findings and a lack of consensus on the exact mechanism of NLRP3 activation. Consequently, multiple upstream signals, possibly acting synergistically or autonomously, are suggested to contribute to NLRP3 inflammasome activation.

The tight regulation of the inflammasome activation process is crucial for an effective immune response and preventing aberrant inflammation.^15^ Dysregulation of the NLRP3-caspase-1-IL-1*β*/IL-18 axis has been implicated in various inflammatory conditions, autoimmune diseases, and infections, highlighting the importance of understanding the intricacies of NLRP3 inflammasome regulation in immune homeostasis.^16^^,^^17^^,^^18^ To grasp the full picture of NLRP3 activation and/or dysregulation, a comprehensive fingerprinting is required. However, NLRP3-related fingerprints are mainly based on molecular profiling approaches that are time and cost inefficient. Concurrently, image-based high-throughput phenotypic profiling is emerging as a promising new approach methodology, providing the opportunity to explore and link phenotypes and modes of action through high-content screen fingerprints.^19^ The Cell Painting assay is a well-established image-based profiling assay that has so far been mostly employed in profiling of pharmacologically and environmentally relevant compounds.^20^^,^^21^ To allow the identification of phenotypic alterations due to chemical or particle exposure, Cell Painting combines multiplexed fluorescent dyes and automated image analysis at single-cell resolution.^22^^,^^23^^,^^24^^,^^25^ By this, the Cell Painting assay holds potential in exploring a broad bioactivity space of NLRP3 triggers in human macrophages without requiring a prior target hypothesis. To our knowledge, Cell Painting has not previously been applied to THP-1 macrophages, nor has it been used in the context of NLRP3 inflammasome activation.

The overarching aim of this study was to identify morphological features in human immune cells in response to NLRP3 inflammasome triggers and to define NLRP3 activity-related phenotypic fingerprints. This was done by investigating the impacts of a selected set of both endogenous and exogenous NLRP3 triggers on the phenotypic profiles of human THP-1 macrophages using the Cell Painting assay. Specifically, the design of the study enabled i) characterization of phenotypic profiles of macrophages following activation with different NLRP3 triggers, ii) assessment of priming effects by using LPS followed by activation with the NLRP3 triggers, and iii) examination of NLRP3-dependency of cellular features by NLRP3 inhibition using MCC950 before activation with triggers. Our findings show that phenotypic profiles can be generated with high reproducibility using THP-1 macrophages. Furthermore, cellular fingerprints containing both common and unique morphological features can be identified for different triggers of inflammasome activation, which are in agreement with previously elucidated modes of action,^26^^,^^27^^,^^28^ highlighting the potential of usage of Cell Painting in inflammasome research.

## Results and discussion

### Selection of exposure levels for inflammasome trigger treatments

**Figure 1:**
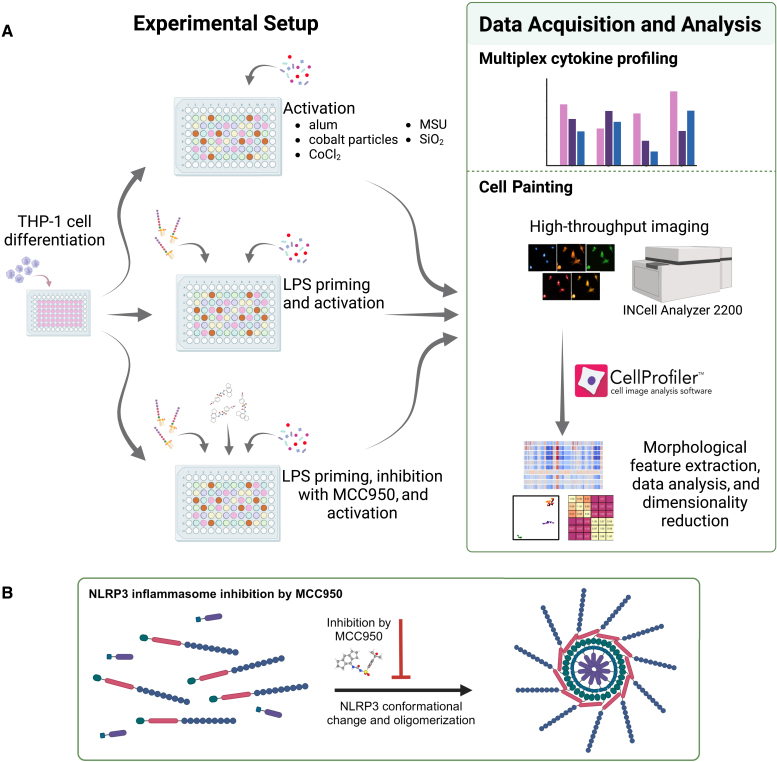
Study overview (A) The human monocyte cell line THP-1 was differentiated into macrophages by the addition of PMA, followed by a 72-h resting period to allow the cells to recover and return to their basal state. NLRP3 inflammasome activation was primed with LPS or mock treated with culture medium before the addition of NLRP3 triggers. Triggers were added with or without prior addition of the NLRP3 inhibitor MCC950, and the cells were incubated for 24 h. The cell medium was collected for multiplex cytokine profiling, and cells were stained for high-throughput imaging and subsequent feature extraction by the CellProfiler software. (B) MCC950 inhibition of NLRP3, a simplified mode of action. The figure was created with BioRender.com.

To investigate morphological features induced by inflammasome triggers, a selection of inflammasome triggers were selected—some commonly used in the field of NLRP3 research (Alum, monosodium urate [MSU], and silica dioxide [SiO_2_])^10^^,^^29^^,^^30^^,^^31^^,^^32^^,^^33^^,^^34^^,^^35^ and some arguably, less frequently used triggers (cobalt particulate and cobalt chloride [CoCl_2_]).^36^^,^^37^^,^^38^ The concentrations chosen for all triggers were based on the manufacturer’s recommendations for inflammasome activation with the respective triggers and published studies, with in-house verification to ensure minimal loss of cells for Cell Painting due to decreased cell viability.^39^^,^^40^ Cobalt nanoparticle exposure levels of 10 μg/mL or higher have been reported to significantly decrease the viability of THP-1 cells over 24 h of exposure,^39^ whereas we found that sufficient numbers of cells remained at a cobalt particulate concentration of 50 μg/mL. The experimental procedure was conducted as outlined in Figure 1.

### High-dimensional phenotypic profiling of NLRP3 triggers

**Figure 2:**
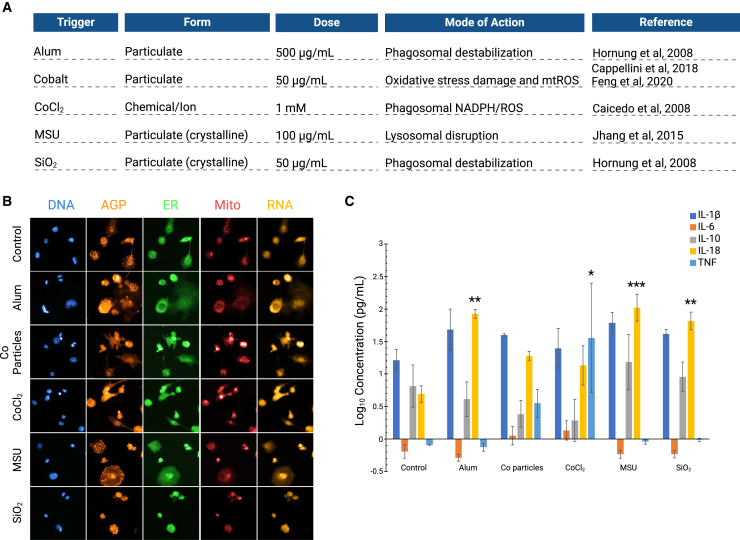
Cell Painting and cytokine profile of triggered human THP-1 macrophages (A) NLRP3 inflammasome triggers (Alum, cobalt [Co] particles, CoCl_2_, MSU, or SiO_2_) employed in the study, and their mode of action according to the published literature. (B) Representative images of THP-1 macrophages exposed to different NLRP3 triggers for 24h before being labeled for the mitochondria (Mito), nuclei (DNA), F-actin/Golgi apparatus/plasma membrane (AGP), endoplasmic reticulum (ER), and RNA/nucleoli (RNA). (C) Extracellular cytokine concentrations were detected in the supernatant of THP-1 macrophages after exposure to various triggers. Results are shown as mean ± SE. Data were analyzed by one-way ANOVA followed by Dunnett multiple comparisons test, ∗*p* <0.05, ∗∗*p* < 0.01, ∗∗∗*p* < 0.001, *n* = 3. The figure was created with BioRender.com.

To generate phenotypic profiles of THP-1 macrophages, the cells were exposed to NLRP3 triggers, with their specific modes of action reported in Figure 2A. Following exposure, the THP-1 macrophages were stained according to the Cell Painting protocol.^22^^,^^24^ Representative images of control cells and cells exposed to NLRP3 triggers are shown in Figure 2B. Currently employed pipelines for high-content imaging and subsequent analysis by CellProfiler developed for U-2 OS or A549 cell lines could not be immediately employed on the THP-1 macrophages due to cell spreading, cell overlap, and clumping behavior. This required optimization of the cell number, particularly because THP-1-differentiated macrophages do not divide.

In addition to the Cell Painting profiling, standard cytokine profiling was performed to assess the ability of the chosen triggers to elicit the secretion of various cytokines, including the NLRP3-related cytokines IL-18 and IL-1β. Exposure to the particulate NLRP3 triggers Alum, MSU, and SiO_2_ induced similar patterns of cytokine release, with a significant increase in the inflammasome/caspase-1-processed cytokine IL-18, but no increase in IL-1β, IL-6, IL-10, or TNF (Figure 2C). Exposure to cobalt particles did not significantly increase the extracellular concentration of any of the cytokines included in the panel. Cobalt chloride, the only non-particulate trigger tested, induced an increase in released TNF that is consistent with previous studies in THP-1 cells.^41^ Canonical NLRP3 inflammasome activation involves two signals: a priming signal and an activation signal, reviewed in Swanson et al., 2019.^42^ The priming signal, commonly induced by LPS in experimental settings, is crucial for upregulating *IL1B* gene expression, among other functions.^43^^,^^44^^,^^45^ Conversely, *IL18* expression is constitutive and does not require a priming signal.^43^^,^^45^ In line with descriptions that priming in THP-1 cells only affects IL-1β,^45^ we find that the NLRP3 inflammasome activators Alum, MSU, and SiO_2_, in the absence of a priming signal, all induce release of IL-18 but not IL-1β.

**Figure 3:**
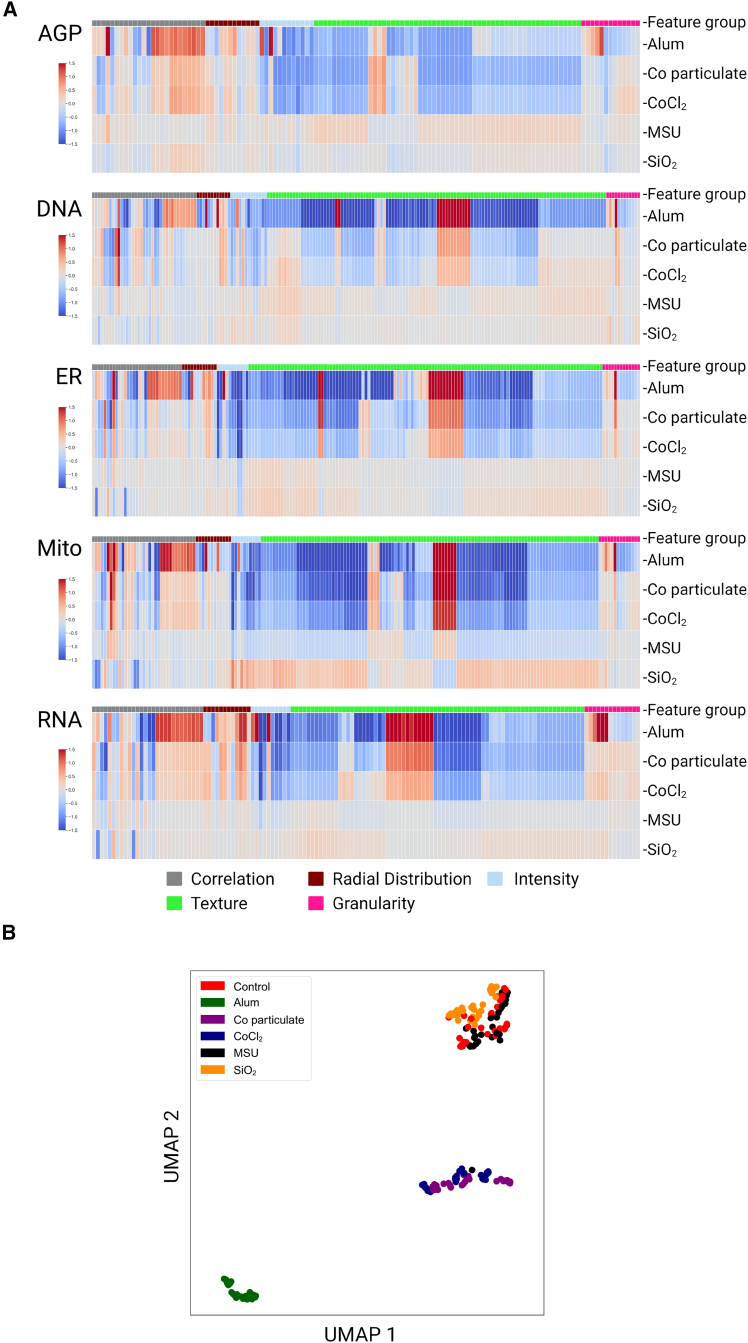
Phenotypic profiling of macrophages exposed to NLRP3 triggers (A) The heatmap was constructed with 779 phenotypic features, retrieved from Cell Painting single-cell profiles of THP-1 macrophages exposed to various triggers. The features represent the *“Cells”* compartment of features extracted using CellProfiler. The feature values are shown at the treatment-level aggregation. The features were grouped into five channels: AGP (121), DNA (194), RNA (138), Mito (185), and ER (189). Each color on the heatmap indicates the fold change, either an increase (red) or decrease (blue), of the respective phenotypic feature compared with the unexposed control. Each column in the heatmap corresponds to a specific phenotypic feature. (B) Uniform Manifold Approximation and Projection (UMAP) was used to project high-dimensional data into a lower-dimensional space at the well-level aggregation of 215,481 single-cell profiles of THP-1 macrophages obtained from ten technical replicate wells across three microplates, constituting biological replicates. For the results presented in the figure, quality control, feature selection procedures, and batch effect correction were performed on the data extracted from these three microplates. Mito, mitochondria; DNA, nuclei; AGP, F-actin/Golgi apparatus/plasma membrane; ER, endoplasmic reticulum; RNA, RNA/nucleoli. The figure was edited in BioRender.com.

Exposure of THP-1 macrophages to NLRP3 triggers induced significant changes in subsets of features, with notable impacts on cellular and subcellular morphology, particularly on mitochondrial measures, including radial distribution, texture, and intensity-related features, as summarized in heatmaps and Uniform Manifold Approximation and Projection (UMAP) in Figures 3 and S1. For example, radial distribution measures the intensity distribution from each object’s center to its boundary within a set of bins. Texture quantifies the degree and nature of textures within images and objects, assessing their roughness and smoothness. Intensity evaluates several intensity features across the entire image. These features have previously been shown to have a high positive predictive value with regards to mitochondrial toxicity,^46^ and mitochondrial dysfunction has a key role in NLRP3 inflammasome activation.^47^ Detailed descriptions of these features can be found in the CellProfiler measurement manual (https://cellprofiler-manual.s3.amazonaws.com/CellProfiler-3.0.0/modules/measurement.html). The top 10 altered features for each trigger are reported in Figure S2.

For Alum (particulate), 1433 of 2584 phenotypic features were significantly altered (55%, *p* < 0.05), emphasizing changes in cellular distribution, texture, and intensity-related features of mitochondria, and in a smaller subset of RNA and DNA features. Exposure to cobalt particles led to changes in 1483 of 2584 features (57%, *p* < 0.05), with a focus on cellular intensity, shape, and texture-related measures. The top altered features indicated significant impacts on mitochondrial and RNA-related parameters, suggesting alterations regarding the mitochondrial structure and RNA content. Exposure to CoCl_2_ (chemical) induced changes in 677 of 2584 features (26%, *p* < 0.05), primarily highlighting modifications in the nuclear compartment. The top 10 altered features emphasized significant impacts on mitochondrial radial distribution, texture, and endoplasmic reticulum (ER) intensity features. Exposure to SiO_2_ (particulate) resulted in significant changes in 547 of 2584 features (21%, *p* < 0.05). The emphasis was on mitochondrial tubeness (a measure of mitochondrial shape, i.e., how “tube-like” the mitochondria appear) and DNA granularity, pointing toward significant impacts on mitochondrial and DNA structure upon SiO_2_ exposure. For MSU (particulate; crystal-like morphology), exposure induced changes in only 61 of 2584 features (2%, *p* < 0.05), particularly related to mitochondrial tubeness and DNA radial distribution. Studies have shown that MSU exposure affects mitochondrial morphology^48^ by increasing levels of dynamin-related protein 1, increasing mitochondrial fission.^27^^,^^28^ The data illustrate the capability of Cell Painting to capture morphological changes that reflect biological modes of action. When examining phenotypic features within the lower dimensional space (illustrated in UMAP in Figure 3B), it becomes apparent that the THP-1 macrophage model provides experimental reproducibility with regard to both imaging and data analysis.

### Cell Painting distinguishes phenotypic fingerprints of different NLRP3 triggers

**Figure 4:**
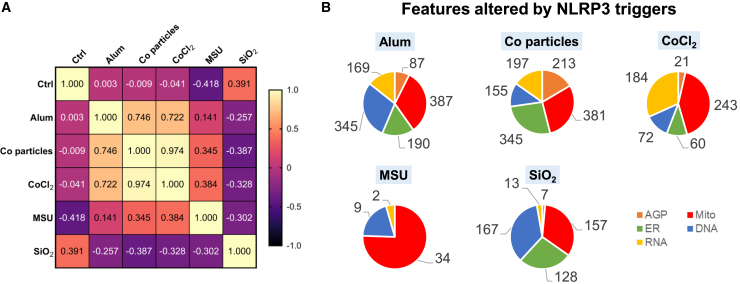
Comparing phenotypic fingerprints of different NLRP3 triggers (A) Correlation matrix comparing average fold change values of Cell Painting phenotypic features of THP-1 macrophages and the calculated Pearson correlation coefficients (R), for control (Ctrl), Alum, cobalt particles, CoCl_2_, MSU, and SiO_2_. (B) Pie charts indicate a relative number of phenotypic features altered by NLRP3 triggers across measured cell compartments of THP-1 macrophages. Mito, mitochondria; DNA, nuclei; AGP, F-actin/Golgi apparatus/plasma membrane; ER, endoplasmic reticulum; RNA, RNA/nucleoli.

In order to assess similarity in phenotypic profiles of different NLRP3 triggers, a correlation analysis was performed. The analysis revealed that phenotypes of THP-1 cells exposed to cobalt particles and CoCl_2_ were highly similar (R = 0.97), as shown in Figure 4A. It has previously been demonstrated that Co nanoparticles release Co^2+^ ions in a concentration- and time-dependent manner,^49^ which likely explains the fact that most of the features observed after triggering with CoCl_2_ were also observed after triggering with cobalt particles. Moreover, a high correlation was found between phenotypic profiles of Alum and cobalt particles and of Alum and CoCl_2_-exposed cells (R = 0.75 and 0.72, respectively). When comparing features related to different compartments, each trigger elicited a specific fingerprint, as summarized in Figure 4B. Mitochondria were a highly affected compartment in the case of all triggers, followed by ER phenotypic measures. The F-actin, Golgi apparatus, and plasma membrane (AGP) features were the least affected, except after triggering with cobalt particles.

### Exploring the impact of NLRP3 priming and inhibition on cell phenotypes and cytokine release

**Figure 5:**
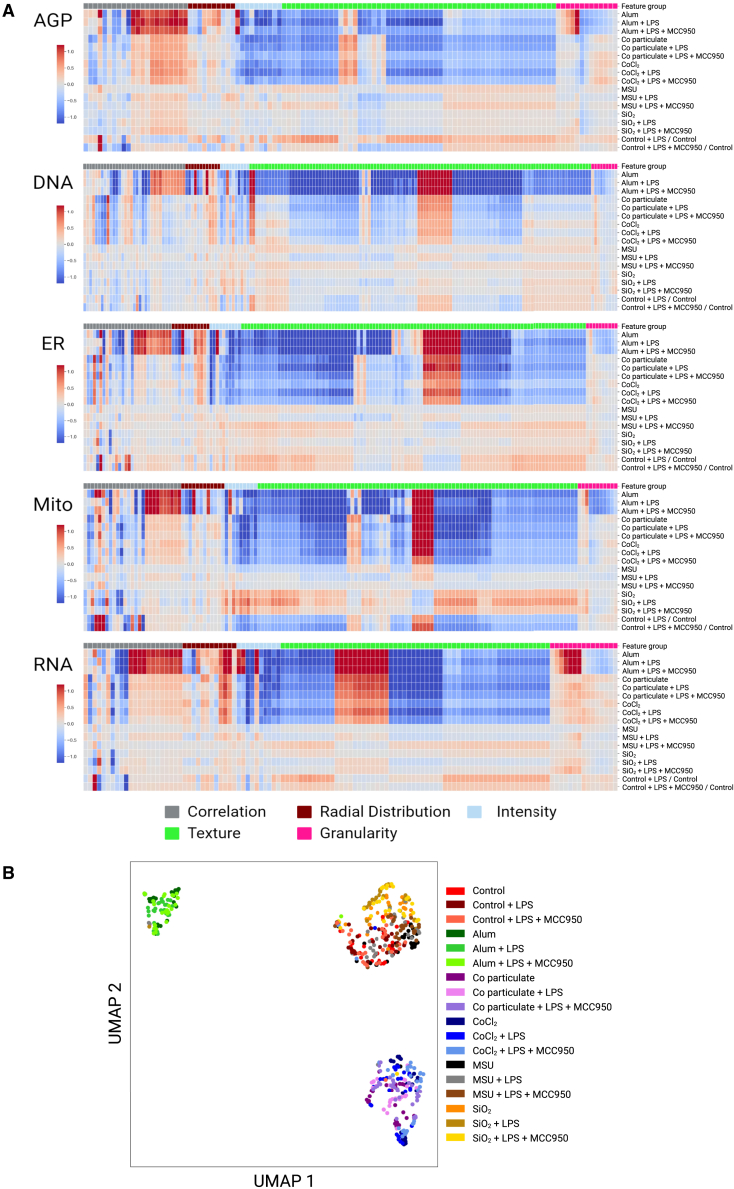
Impact of NLRP3 priming and inhibition on cell phenotypes (A) A heatmap depicting 696 phenotypic features, representing the *“Cells”* compartment, categorized into five channels: AGP (113), DNA (184), RNA (119), Mito (148), and ER (170). Colors on the heatmap represent the fold change in the corresponding phenotypic feature after normalization, compared with the unexposed control, with red indicating an increase and blue indicating a decrease. Each column in the heatmap corresponds to a distinct phenotypic feature. (B) Uniform Manifold Approximation and Projection (UMAP) of high-dimensional data into a lower-dimensional space at the well-level aggregation of cell profiles. The dataset comprised 554,981 single-cell profiles of THP-1 macrophages collected from ten technical replicate wells and three microplates, encompassing biological replicates. For the results presented in the figure, quality control, feature selection procedures, and batch effect correction were performed on the data extracted from the whole dataset. Mito, mitochondria; DNA, nuclei; AGP, F-actin/Golgi apparatus/plasma membrane; ER, endoplasmic reticulum; RNA, RNA/nucleoli.

**Figure 6:**
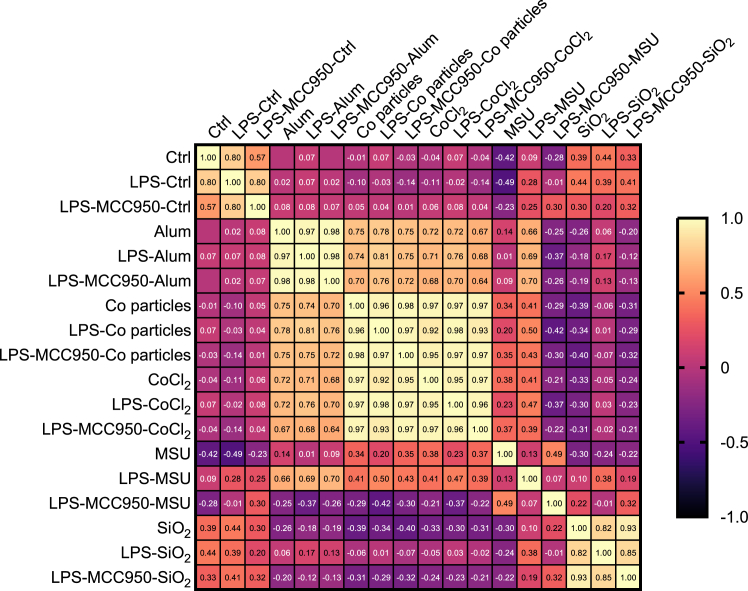
Comparison of Cell Painting phenotypic fingerprints Correlation matrix comparing average fold change values of Cell Painting phenotypic features of THP-1 macrophages and the calculated Pearson correlation coefficients (R), for control (Ctrl), Alum, cobalt (Co) particles, CoCl_2_, MSU, and SiO2 under all exposure conditions (i.e., trigger, LPS-trigger, LPS-MCC950-trigger).

In order to understand the effects of transcriptional upregulation of NLRP3 and pro-IL-1β expression on the cell phenotypic profiles, THP-1 cells were primed with LPS. Interestingly, priming evoked transcriptional reprogramming of cells as observed by extensive changes in the phenotypic features related to texture of RNA and DNA across all triggers (Figure S3). The number of features that were significantly altered upon LPS priming was increased for each trigger, except for SiO_2_ that displayed a decreased number of significantly altered features. However, this approach was not able to discern which features were driven by NLRP3-dependent mechanisms and which were not. Therefore, the effect of NLRP3 inhibition by MCC950 was studied. MCC950 is a sulfonylurea-containing small molecule inhibitor of NLRP3,^50^ and it inhibits the NLRP3 conformational change and oligomerization (Figure 1B) by directly targeting the NLRP3 NACHT-domain and interfering with the Walker B motif function.^51^^,^^52^ Figures 5A and 5B summarize the effects of NLRP3-priming and NLRP3-priming and inhibition on cell phenotypic profiles. When evaluating all the features summarized in Figures 5A and S4, including phenotypic profiles of cells exposed to the respective trigger; LPS and trigger; or LPS, inhibitor, and trigger, it was difficult to discern if NLRP3 inhibition was able to revert specific NLRP3-related phenotypic features to their baseline state. However, there was a general trend indicating that the phenotypic profiles of cells exposed to the respective NLRP3 trigger or LPS, inhibitor, and trigger displayed a closer phenotypic resemblance to each other compared with the profiles of cells primed with LPS and then exposed to a trigger. This observation indicates that NLRP3 inhibition may mitigate some of the phenotypic alterations induced by NLRP3 activation, although not necessarily restoring features to baseline with these experimental conditions. To further investigate this trend, correlation analysis was conducted, the results of which are reported in Figure 6.

Overall, the phenotypic profiles, observed under different exposure conditions (i.e., activation, priming, inhibition), suggest the involvement of mechanisms associated with NLRP3 activation, as highlighted in Figure 2A. Notably, phagosomal destabilization (Alum and SiO_2_), lysosomal disruption (MSU), and oxidative stress (cobalt particles and CoCl_2_) can lead to the observed phenotypic changes. The oxidative environment impairs mitochondrial function, resulting in altered mitochondrial morphology such as swelling and fragmentation. Concurrently, the ER may undergo stress-induced structural changes, including dilation and altered connectivity, in response to oxidative damage and disrupted cellular homeostasis.^24^

**Figure 7:**
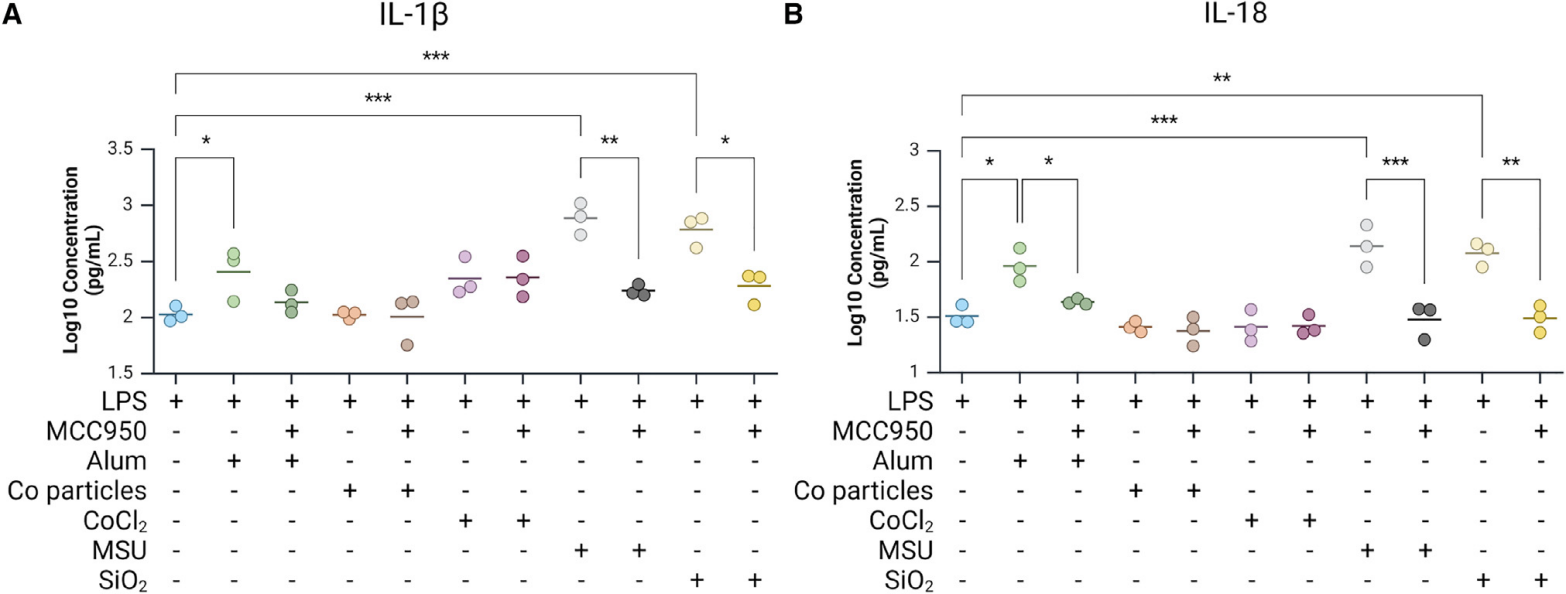
Cytokine release Levels of extracellular (A) IL-1β and (B) IL-18 from THP-1 macrophages following lipopolysaccharide (LPS) priming alone; LPS priming followed by exposure to Alum, cobalt particulate, CoCl_2_, MSU, or SiO_2_; or LPS priming and MCC950 inhibition, followed by exposure to Alum, cobalt particulate, CoCl_2_, MSU, or SiO_2_. Significance for LPS is compared with the control, while significance for the remaining treatments without MCC950 is compared with LPS. The significance for treatments with MCC950 is compared with the equivalent trigger with LPS but without MCC950. Comparisons between treatments without MCC950 and LPS were analyzed by one-way ANOVA with Dunnett multiple comparisons test. The remaining data were analyzed by t test followed by Holm-Šídák multiple comparisons test, ∗*p* < 0.05, ∗∗*p* < 0.01, ∗∗∗<0.001. Figure was created with BioRender.com.

To validate the results given by the NLRP3 inhibition, cytokine release was measured in LPS-primed cells with or without prior addition of MCC950 and the subsequent exposure to the various triggers. Treatment with LPS alone significantly increased the release of the cytokines IL-1β, IL-6, IL-18, and TNF compared with unprimed controls but had no impact on IL-10 release (Figures 7 and S5). Exposure to cobalt particles or CoCl_2_ after priming with LPS did not add further release of cytokine over LPS alone on any of the evaluated cytokines. The addition of Alum, as well as the well-known NLRP3 triggers MSU and SiO_2_, significantly increased IL-1β and IL-18 release after LPS-priming as compared with LPS alone (Figure 7) but had no additional effect on IL-6, IL-10, or TNF release.

The efficacy of blocking NLRP3 by the inhibitor MCC950 was evaluated in terms of cytokine release. Pre-treatment with MCC950 significantly decreased IL-1β release by LPS-primed cells triggered with MSU or SiO_2_ (Figure 7A) and decreased IL-18 release by LPS-primed cells triggered with Alum, MSU, or SiO_2_ (Figure 7B), confirming the role of these triggers as NLRP3 inflammasome activators. MCC950 inhibition of NLRP3 had no effect on IL-6, IL-10, or TNF release by LPS-primed cells triggered with Alum, cobalt particulate, CoCl_2_, MSU, or SiO_2_ (Figures 7 and S5). Reports regarding the ability of CoCl_2_ to activate the NLRP3 inflammasome are contradictory. Cobalt chloride has been reported to induce caspase-1 activation and IL-1β/IL-18 release in keratinocytes^37^ and IL-1β in THP-1 cells^53^ in a dose-dependent manner. Contrary to this, however, CoCl_2_ has also been reported to have no effect in PMA-differentiated THP-1 cells or mouse bone marrow macrophages and to inhibit NLRP3 inflammasome activation in glial cells.^54^ Our data suggest that, at the concentration used here, CoCl_2_ does not induce inflammasome function, i.e., IL-1β and IL-18 release, in PMA-differentiated THP-1 cells.

### Identifying the NLRP3-related phenotypic features

**Figure 8:**
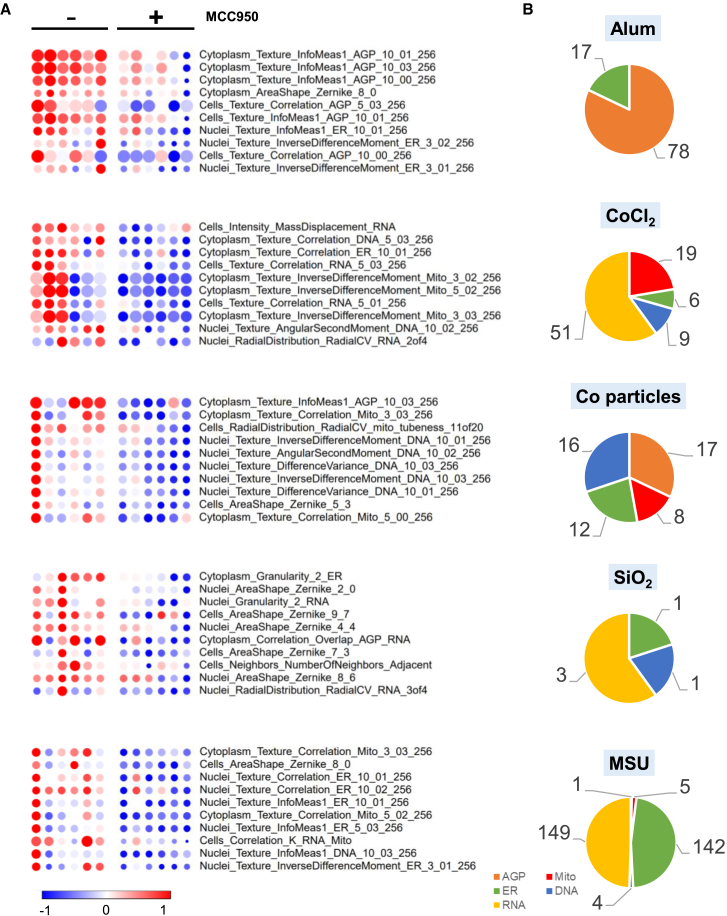
Identifying NLRP3-related morphological features in THP-1 macrophages (A) Heatmaps presenting the top ten phenotypic features that exhibited significant differences (*p* < 0.05) between THP-1 macrophages exposed to the same NLRP3 trigger, with either lipopolysaccharide (LPS) priming alone or LPS priming followed by MCC950 treatment. Colors on the heatmap denote the fold change in features relative to the corresponding control, either THP-1 macrophages following only LPS priming (−) or macrophages subjected to LPS priming and MCC950 inhibition (+). Statistical significance was determined using t test. (B) Pie charts indicate a relative number of phenotypic features for each NLRP3 trigger significantly different between macrophages that were either primed with LPS or primed with LPS followed by MCC950 treatment. Mito, mitochondria; DNA, nuclei; AGP, F-actin/Golgi apparatus/plasma membrane; ER, endoplasmic reticulum; RNA, RNA/nucleoli.

To delineate NLRP3-related phenotypic features, we compared cell profiles of THP-1 macrophages exposed to specific triggers following LPS priming as well as LPS priming coupled with MCC950 inhibition. Phenotypic features that were absent after inhibition of NLRP3 inflammasome formation by MCC950 were deemed as NLRP3-related features. The top ten NLRP3-related phenotypic features for each trigger are illustrated in Figure 8A. At the individual feature level, Alum revealed 102 of 2584 (3.9%) phenotypic features to be NLRP3-related, cobalt particles showed 64 (2.5%), CoCl_2_ had 97 (3.8%), SiO_2_ exhibited 18 (0.7%), and MSU displayed 321 (12.4%). When comparing features related to different cell compartments, each NLRP3 trigger induced a specific phenotypic fingerprint, as summarized in Figure 8B. Interestingly, in the case of Alum, all NLRP3-related phenotypic features were associated with AGP and ER compartments. For CoCl_2_, SiO_2_, and MSU, NLRP3-related features were primarily various RNA measures. Notably, each trigger induced changes in a subset of the ER-related features, making it a unique phenotypic trait for each of the NLRP3 triggers used in this study.

Phenotypic features associated with NLRP3 in the case of Alum were predominantly linked to AGP measures followed by ER-related characteristics. For example, the feature cluster *Cytoplasm_Texture_InfoMeas1_AGP* appears to be NLRP3-related. Notably, Texture_InfoMeas1 features measure the total amount of information contained within a region of pixels in the AGP compartment, mainly providing information on the cytoskeletal structure. As such, these features have recently been found to be associated with cytoskeletal reorganization and ER stress/unfolded protein response.^55^ Importantly, ER stress has previously been reported to activate the NLRP3 inflammasome in macrophages,^56^ which aligns with the Cell Painting findings presented here. Additionally, Burger et al. (2016)^57^ demonstrated that F-actin dampens NLRP3 activity, revealing a regulatory role in NLRP3 inflammasome function. For CoCl_2_, NLRP3-related features were mainly attributed to RNA, followed by mitochondrial measures. In the case of cobalt particles, NLRP3-related features were dispersed across various subcellular structures, with the exception of RNA. Interestingly, both SiO_2_ and MSU demonstrated that NLRP3-related features were RNA-associated, despite a nearly 18-fold difference in total feature counts. Moreover, in the case of MSU, the second most impacted group of NLRP3-related features pertained to ER-related measures. Three of the five triggers (CoCl_2_, SiO_2_, and MSU) demonstrated that RNA-related features were primarily associated with NLRP3. This finding was not unexpected, considering that NLRP3 priming and activation has been shown to substantially remodel the transcriptomic landscape in macrophages.^26^

In conclusion, we find that THP-1 macrophages can be readily employed in Cell Painting and as such provide a powerful cell model for expanding the Cell Painting platform into studies on the cellular functions of innate immune cells. Our described screening strategies indicate that Cell Painting identifies specific cellular fingerprints associated with intricate cellular mechanisms including NLRP3 inflammasome activation. By using a diverse approach of several NLRP3 triggers in combination with NLRP3 priming and inhibition strategies, the use of Cell Painting identifies several unique phenotypic traits of NLRP3 specific activation, with ER-related features standing out as a common strong denominator for all triggers used. The relation between ER stress and NLRP3 inflammasome has previously been described by others providing verification of the usability of Cell Painting in the search for NLRP3 inflammasome-associated phenotypic fingerprinting.

### Limitations of the study

One limitation of this study is the lack of a concurrent assay for inflammasome activation. Activation of the inflammasome in a cell population elicits a heterologous response where individual cells respond at different time points, or not at all,^58^ which may depend on cell-intrinsic features (e.g., metabolic state, expression levels, and signaling readiness) or experimental conditions (e.g., priming efficiency and cell density). Consequently, the features observed in our study are derived from a mix of non-activated cells and activated cells at variable stages post inflammasome activation. It remains unclear how the timing of inflammasome activation influences cell morphology, with a risk that this may influence the data. Inclusion of a marker for inflammasome formation or activation is difficult, as Cell Painting requires the use of five fluorophores and is thus difficult to combine with assays quantifying inflammasome assembly, such as ASC-GFP, or inflammasome activation, such as FAM-FLICA, without removing one or more channels, leading to a significant loss of data. The lack of a robust inflammasome response with all triggers is also a limitation. While we have used concentrations obtained from the literature and/or manufacturer’s recommendations, a strong response is not evidenced with all triggers. Inflammasome formation has, however, been confirmed in the reporter THP1-ASC-GFP cell line for multiple triggers. High concentrations of NLRP3 inflammasome triggers may exaggerate NLRP3 activation, in turn leading to high levels of cell death. With the concentrations utilized in this study, we obtained clear NLRP3-dependent features even when cytokine readouts for inflammasome activation were low.

## Resource availability

### Lead contact

Requests for further information and resources should be directed to and will be fulfilled by the Lead Contact, Andi Alijagic (andi.alijagic@oru.se).

### Materials availability

This study did not generate new unique reagents. Further information and requests for resources and reagents should be directed to and will be fulfilled by the lead contact, Andi Alijagic (andi.alijagic@oru.se).

### Data and code availability

• The treatment level cell profiles and UMAP embeddings data for Figures 3, 5, S1, and S4 are publicly available at https://git.oru.se/ai-tox-med/cell-painting-inflammasome-triggers.
• The code for Figures 3, 5, S1, and S4 is publicly available at https://git.oru.se/ai-tox-med/cell-painting-inflammasome-triggers.
• Any additional information required to reanalyze the data reported in this paper is available from the lead contact upon request.

## Acknowledgments

This work was supported by the Swedish Knowledge Foundation (Grants No. 2016-0044; 2022-0122; 2023-0020). We acknowledge scientific support from the Exploring Inflammation in Health and Disease (X-HiDE) Consortium, which is a strategic research profile at Örebro University funded by the Knowledge Foundation (Grant No. 2020-0017). The computations/data handling were partially enabled by resources provided by the National Academic Infrastructure for Supercomputing in Sweden (NAISS) and the Swedish National Infrastructure for Computing (SNIC) partially funded by the Swedish Research Council (Grant No. 2022-06725 and 2018-05973), projects SNIC 2022/5-535, SNIC 2022/6-306, NAISS 2023/5-511, and NAISS 2023/6-342.

## Author contributions

Conceptualization, E.S., A.P., and A.A.; Data curation, M.H., O.K., and A.A.; Formal analysis, M.H. and A.A.; Funding acquisition, E.S., M. Ejdebäck, A.P., and A.A.; Investigation, M.H., A.P., and A.A.; Methodology, M.H., O.K., and A.A.; Project administration, E.S., M. Ejdebäck, and A.A.; Resources, M.H., O.K., M. Engwall, and A.A.; Software, M.H., O.K., and A.A.; Supervision, E.S., R.K., M. Ejdebäck, A.P., and A.A.; Validation, M.H., E.S., O.K., N.S., M. Engwall, R.K., M. Ejdebäck, A.P., and A.A.; Visualization: M.H. and A.A.; Writing – original draft, M.H. and A.A.; Writing – review & editing, M.H., E.S., O.K., N.S., M. Engwall, R.K., M. Ejdebäck, A.P., and A.A.

## Declaration of interests

R.K. is affiliated to the company Nanoxis Consulting AB. The company did not have any influence on the conception, analysis or interpretation of data, the writing of the paper, or the decision to submit the present manuscript for publication.

## STAR★Methods

### Key resources table

**Table:** 

REAGENT or RESOURCE	SOURCE	IDENTIFIER
**Chemicals, peptides, and recombinant proteins**		

MitoTracker™	Invitrogen	Cat#M22426
Phalloidin/Alexa Fluor 568	Invitrogen	Cat#A12380
Concanavalin A/Alexa Fluor 488	Invitrogen	Cat#C11252
Hoechst	Thermo Fisher Scientific	Cat#H3570
wheat germ agglutinate-Alexa Fluor™ 555 conjugate	Invitrogen	Cat#W32464
SYTO™ 14	Invitrogen	Cat#S7576
MCC950	InvivoGen	Cat. Code:inh.mcc
Alum	InvivoGen	Cat. Code:tlrl-aloh
Cobalt particles	Sigma-Aldrich	Cat# 266639
Cobalt chloride	Sigma-Aldrich	Cat#15862-1ML-F
MSU	InvivoGen	Cat. Code:tlrl-msu
Silica (SiO2)	InvivoGen	Cat. Code:tlrl-sio-2
RPMI-1640	Fisher Scientific	Cat# A1049101
FBS	Biowest	Cat#S181H
Penicillin-streptomycin	Sigma-Aldrich	Cat#P4333

**Critical commercial assays**		

LEGENDplex™ Multi-Analyte Flow Assay Kit, Human Inflammation panel 1	BioLegend	Cat#740809

**Deposited data**		

Code for plotting Figures 3 and S1	This Paper	https://git.oru.se/ai-tox-med/cell-painting-inflammasome-triggers/-/blob/master/Figure 3_and_S1/heatmaps_umap_fig3_s1.ipynb
Code for plotting Figures 5 and S4	This Paper	https://git.oru.se/ai-tox-med/cell-painting-inflammasome-triggers/-/blob/master/Figure 5_and_S4/heatmaps_umap_fig5_s4.ipynb
Data for Figures 3A and S1	This Paper	https://git.oru.se/ai-tox-med/cell-painting-inflammasome-triggers/-/blob/master/Figure 3_and_S1/inflammasome_triggers_THP1_well_level.csv
Data for Figure 3B	This Paper	https://git.oru.se/ai-tox-med/cell-painting-inflammasome-triggers/-/blob/master/Fig3_and_S1/UMAP_data_inflammasome_triggers_THP1.csv
Data for Figures 5A and S4	This Paper	https://git.oru.se/ai-tox-med/cell-painting-inflammasome-triggers/-/blob/master/Fig5_and_S4/inflammasome_triggers_THP1_treatment_level_ctrl_LPS_MCC950_ext_norm.csv https://git.oru.se/ai-tox-med/cell-painting-inflammasome-triggers/-/blob/master/Fig5_and_S4/inflammasome_triggers_THP1_well_level_all_conditions.csv
Data for Figure 5B	This Paper	https://git.oru.se/ai-tox-med/cell-painting-inflammasome-triggers/-/blob/master/Fig5_and_S4/UMAP_data_inflammasome_triggers_THP1_well_level_all_conditions.csv

**Experimental models: Cell lines**		

THP1	InvivoGen	Cat. Code: thp-null

**Software and algorithms**		

LEGENDplex™ Data Analysis Software	https://www.biolegend.com/en-gb/immunoassays/legendplex/support/software	
CellProfiler	https://cellprofiler.org/	
MetaboAnalyst	www.metaboanalyst.ca^59^	
Morpheus	https://software.broadinstitute.org/morpheus	
GraphPad Prism v. 10.4.0.	https://www.graphpad.com/scientific-software/prism/www.graphpad.com/scientific-software/prism/	

### Experimental model and subject details

#### Cell culture

THP-1 cells (InvivoGen, San Diego, Ca, USA), a human (male) peripheral blood monocyte cell line, were cultured in RPMI 1640 (Gibco; Thermo Fisher Scientific, Waltham, MA, USA) supplemented with 10% heat-inactivated FBS (Biowest, Bionordic A/S, Søborg, Demark) and 100 U/mL penicillin-streptomycin (Sigma-Aldrich; Merck, Darmstadt, Germany) at 37°C, 5% CO_2_ in a humidified incubator. Cells were passaged every 2–3 days as required to maintain a cell density between 6x10^5^ and 1.5x10^6^ cells/mL, up to passage number ten, after which they were discarded.

### Method details

#### Cell exposure

THP-1 cells were seeded and differentiated in 96-well imaging plates (Sarsdedt, Nümbrecht, Germany) at 3x10^4^ cells/well in 200 μL of complete media containing 100 nM phorbol 12-myristate 13-acetate (PMA; InvivoGen) for 72 h. To the outer wells of the plate 200 μL PBS was added to minimize edge effects. Cells were subsequently washed three times with fresh media and rested for 48 h at 37°C, 5% CO_2_ in a humidified incubator. Cells were primed with a final concentration of 100 ng/mL ultrapure LPS (InvivoGen) for 3 h in fresh media while unprimed cells received only fresh media, followed by washing with fresh media three times for both treatments. Cells were then treated with 3 μM MCC950 (inhibited; InvivoGen) or the equivalent volume of culture media (uninhibited) for 1 h followed by the addition of either 500 μg/mL aluminum hydroxide, 50 μg/mL cobalt particulate, 1 mM CoCl_2_, 100 μg/mL MSU (InvivoGen), 50 μg/mL SiO_2_ (InvivoGen) or media (control) and incubated for 24 h at 37°C, 5% CO_2_ in a humidified incubator. All concentrations listed are final concentrations. Five technical replicates were included for each stimulus, and replicate locations were randomly allocated within each plate to minimize plate position effects.

Trigger concentrations were chosen based on the literature or respective manufacturer’s recommendations. Cytotoxic effects, such as rounding, changes in cell size and cell density, were assessed by microscopic inspection. In addition, the number of remaining cells was quantified using CellProfiler to count viable nuclei (stained with Hoechst 33342) in all controls (non-treated, LPS-treated and LPS + MCC950-treated), as well as after exposure to triggers.

#### Cytokine profiling

After triggering for 24 h, cell media was removed and stored at −20°C for cytokine profiling. Samples were thawed on ice and technical replicates were pooled and analyzed with the LEGENDplex Multi-Analyte Flow Assay Kit, Human Inflammation panel 1 (BioLegend, San Diego, CA, USA) for V-bottom plates as instructed using a Gallios flow cytometer (Beckman Coulter, Brea, CA, USA).

#### Cell Painting-staining and image acquisition

Cell Painting assay was performed as previously described by Bray et al. (2016),^22^ with slight modifications.^24^ Pre-warmed cell media containing 0.5 μM MitoTracker Red (Invitrogen; Thermo Fisher Scientific, Eugene, OR, USA) was added to each well and plates were incubated for 30 min at 37°C, 5% CO_2_ in a humidified incubator. Paraformaldehyde was added to a final concentration of 4% followed by 20 min incubation in the dark at room temperature. Wells were emptied by inverting the plates, followed by washing two times with PBS. Cells were subsequently permeabilized and stained by the addition of 30 μL of prewarmed staining solution consisting of 8.25 nM Phalloidin/Alexa Fluor 568 (Invitrogen), 5 μg/mL Concanavalin A/Alexa Fluor 488 (Invitrogen), 1 μg/mL Hoechst (Thermo Fisher Scientific), 1.5 μg/mL wheat germ agglutinate-Alexa Fluor 555 conjugate (Invitrogen), 6 μM SYTO 14 (Invitrogen), 0.1% Triton X- and 0.1% BSA in PBS, and incubated for 30 min at RT in the dark. Wells were washed six times with PBS and stored at 4°C until imaged. The high-throughput imaging platform InCell 2200 HTS system, (GE Healthcare; Uppsala, Sweden), was employed for image acquisition.

### Quantification and statistical analysis

#### Cytokine profiling

Cytokine data was analyzed using the LEGENDplex Data Analysis Software (BioLegend). Due to the non-normal nature of the data, cytokine concentrations were log_10_ transformed. Statistical analysis was performed using MetaboAnalyst.^59^ Data is shown as mean or mean ± SEM. Data were analyzed by one-way ANOVA followed by Dunnett multiple comparisons test or t-test followed by Holm-Šídák multiple comparisons test, as specified.

#### Image analysis, profiling, and dimensionality reduction

CellProfiler v. 4.2.1, an open-source image analysis software (www.cellprofiler.org),^60^ was utilized to extract morphological features. The analysis involved running illumination correction (JUMP_illum_LoadData_v1.cppipe) and analytical (JUMP_analysis_v3.cppipe) pipelines developed by the Broad Institute for the JUMP – Cell Painting Consortium (https://github.com/broadinstitute/imaging-platform-pipe lines/tree/master/JUMP_production). Post-analysis, 3,676 feature measurements were extracted for each cell profile from 554,981 control and exposed cells.

Following feature extraction, quality control procedures, normalization, averaging to the treatment level values, feature selection, and batch effect correction were implemented, as described in Alijagic et al. (2023)^24^ and Alijagic et al. (2024).^61^

Normalization against unexposed control cells and averaging of the single-cell profile values to the treatment level values were performed using an algorithm described by Bray et al. (2016).^22^ During the quality control step, fields-of-view with fewer than 5 cells and wells with fewer than a total of 20 cells were excluded from further analysis. Feature selection excluded noisy features using an algorithm similar to Pycytominer’s (https://github.com/cytomining/pycytominer), and features with absolute extreme values larger than 500 were excluded. Batch effect correction was executed using pyComBat software.^62^ MORPHEUS (https://software.broadinstitute.org/morpheus/), an online analysis software, was used for profiling phenotypes and detection of significantly changed features by t-test.

These procedures resulted in 2,584 feature measurements for further analysis. The approach allowed a comparison of morphological profiles between control and exposed cells. Heatmaps were constructed using self-developed Python scripts, available upon request. To visualize high-dimensional data in a lower-dimensional space, Uniform Manifold Approximation and Projection (UMAP)^63^ was applied, displaying the data in a 2-dimensional UMAP space.

## References

[1] Liu X., Zhang Z., Ruan J., Pan Y., Magupalli V.G., Wu H., Lieberman J. (2016). Inflammasome-activated gasdermin D causes pyroptosis by forming membrane pores. Nature.

[2] Fu J., Wu H. (2023). Structural Mechanisms of NLRP3 Inflammasome Assembly and Activation. Annu. Rev. Immunol..

[3] Seok J.K., Kang H.C., Cho Y.Y., Lee H.S., Lee J.Y. (2020). Regulation of the NLRP3 Inflammasome by Post-Translational Modifications and Small Molecules. Front. Immunol..

[4] Bauernfeind F.G., Horvath G., Stutz A., Alnemri E.S., MacDonald K., Speert D., Fernandes-Alnemri T., Wu J., Monks B.G., Fitzgerald K.A. (2009). Cutting edge: NF-kappaB activating pattern recognition and cytokine receptors license NLRP3 inflammasome activation by regulating NLRP3 expression. J. Immunol..

[5] Netea M.G., Nold-Petry C.A., Nold M.F., Joosten L.A.B., Opitz B., van der Meer J.H.M., van de Veerdonk F.L., Ferwerda G., Heinhuis B., Devesa I. (2009). Differential requirement for the activation of the inflammasome for processing and release of IL-1beta in monocytes and macrophages. Blood.

[6] Perregaux D., Gabel C.A. (1994). Interleukin-1 beta maturation and release in response to ATP and nigericin. Evidence that potassium depletion mediated by these agents is a necessary and common feature of their activity. J. Biol. Chem..

[7] Dostert C., Pétrilli V., Van Bruggen R., Steele C., Mossman B.T., Tschopp J. (2008). Innate immune activation through Nalp3 inflammasome sensing of asbestos and silica. Science (New York, N.Y.).

[8] Zhou R., Yazdi A.S., Menu P., Tschopp J. (2011). A role for mitochondria in NLRP3 inflammasome activation. Nature.

[9] Lee G.S., Subramanian N., Kim A.I., Aksentijevich I., Goldbach-Mansky R., Sacks D.B., Germain R.N., Kastner D.L., Chae J.J. (2012). The calcium-sensing receptor regulates the NLRP3 inflammasome through Ca2+ and cAMP. Nature.

[10] Hornung V., Bauernfeind F., Halle A., Samstad E.O., Kono H., Rock K.L., Fitzgerald K.A., Latz E. (2008). Silica crystals and aluminum salts activate the NALP3 inflammasome through phagosomal destabilization. Nat. Immunol..

[11] Iyer S.S., He Q., Janczy J.R., Elliott E.I., Zhong Z., Olivier A.K., Sadler J.J., Knepper-Adrian V., Han R., Qiao L. (2013). Mitochondrial cardiolipin is required for Nlrp3 inflammasome activation. Immunity.

[12] Muñoz-Planillo R., Kuffa P., Martínez-Colón G., Smith B.L., Rajendiran T.M., Núñez G. (2013). K⁺ efflux is the common trigger of NLRP3 inflammasome activation by bacterial toxins and particulate matter. Immunity.

[13] Chen J., Chen Z.J. (2018). PtdIns4P on dispersed trans-Golgi network mediates NLRP3 inflammasome activation. Nature.

[14] Alijagic A., Hedbrant A., Persson A., Larsson M., Engwall M., Särndahl E. (2023). NLRP3 inflammasome as a sensor of micro- and nanoplastics immunotoxicity. Front. Immunol..

[15] Paik S., Kim J.K., Silwal P., Sasakawa C., Jo E.K. (2021). An update on the regulatory mechanisms of NLRP3 inflammasome activation. Cell. Mol. Immunol..

[16] Mangan M.S.J., Olhava E.J., Roush W.R., Seidel H.M., Glick G.D., Latz E. (2018). Targeting the NLRP3 inflammasome in inflammatory diseases. Nat. Rev. Drug Discov..

[17] Zhong Y., Lu Y., Yang X., Tang Y., Zhao K., Yuan C., Zhong X. (2020). The roles of NLRP3 inflammasome in bacterial infection. Mol. Immunol..

[18] Zhang Y., Yang W., Li W., Zhao Y. (2021). NLRP3 Inflammasome: Checkpoint Connecting Innate and Adaptive Immunity in Autoimmune Diseases. Front. Immunol..

[19] Severin Y., Hale B.D., Mena J., Goslings D., Frey B.M., Snijder B. (2022). Multiplexed high-throughput immune cell imaging reveals molecular health-associated phenotypes. Sci. Adv..

[20] Nyffeler J., Willis C., Lougee R., Richard A., Paul-Friedman K., Harrill J.A. (2020). Bioactivity screening of environmental chemicals using imaging-based high-throughput phenotypic profiling. Toxicol. Appl. Pharmacol..

[21] Rietdijk J., Tampere M., Pettke A., Georgiev P., Lapins M., Warpman-Berglund U., Spjuth O., Puumalainen M.-R., Carreras-Puigvert J. (2021). A phenomics approach for antiviral drug discovery. BMC Biol..

[22] Bray M.A., Singh S., Han H., Davis C.T., Borgeson B., Hartland C., Kost-Alimova M., Gustafsdottir S.M., Gibson C.C., Carpenter A.E. (2016). Cell Painting, a high-content image-based assay for morphological profiling using multiplexed fluorescent dyes. Nat. Protoc..

[23] Pahl A., Schölermann B., Lampe P., Rusch M., Dow M., Hedberg C., Nelson A., Sievers S., Waldmann H., Ziegler S. (2023). Morphological subprofile analysis for bioactivity annotation of small molecules. Cell Chem. Biol..

[24] Alijagic A., Scherbak N., Kotlyar O., Karlsson P., Wang X., Odnevall I., Benada O., Amiryousefi A., Andersson L., Persson A. (2023). A Novel Nanosafety Approach Using Cell Painting, Metabolomics, and Lipidomics Captures the Cellular and Molecular Phenotypes Induced by the Unintentionally Formed Metal-Based (Nano)Particles. Cells.

[25] Seal S., Trapotsi M.-A., Spjuth O., Singh S., Carreras-Puigvert J., Greene N., Bender A., Carpenter A.E. (2024). A Decade in a Systematic Review: The Evolution and Impact of Cell Painting. bioRxiv.

[26] Zuo Z., Shi J., Wang Y., Yin Z., Wang Z., Yang Z., Jia B., Sun Y. (2024). The transcriptomic landscape of canonical activation of NLRP3 inflammasome from bone marrow-derived macrophages. Biochem. Biophys. Res. Commun..

[27] Lavie J., Serrat R., Bellance N., Courtand G., Dupuy J.-W., Tesson C., Coupry I., Brice A., Lacombe D., Durr A. (2017). Mitochondrial morphology and cellular distribution are altered in SPG31 patients and are linked to DRP1 hyperphosphorylation. Hum. Mol. Genet..

[28] Jiang H., Chen F., Song D., Zhou X., Ren L., Zeng M. (2022). Dynamin-Related Protein 1 Is Involved in Mitochondrial Damage, Defective Mitophagy, and NLRP3 Inflammasome Activation Induced by MSU Crystals. Oxid. Med. Cell. Longev..

[29] Kool M., Pétrilli V., De Smedt T., Rolaz A., Hammad H., van Nimwegen M., Bergen I.M., Castillo R., Lambrecht B.N., Tschopp J. (2008). Cutting edge: alum adjuvant stimulates inflammatory dendritic cells through activation of the NALP3 inflammasome. J. Immunol..

[30] Eisenbarth S.C., Colegio O.R., O'Connor W., Sutterwala F.S., Flavell R.A. (2008). Crucial role for the Nalp3 inflammasome in the immunostimulatory properties of aluminium adjuvants. Nature.

[31] Li H., Willingham S.B., Ting J.P.Y., Re F. (2008). Cutting edge: inflammasome activation by alum and alum's adjuvant effect are mediated by NLRP3. J. Immunol..

[32] Franchi L., Núñez G. (2008). The Nlrp3 inflammasome is critical for aluminium hydroxide-mediated IL-1beta secretion but dispensable for adjuvant activity. Eur. J. Immunol..

[33] Piancone F., Saresella M., Marventano I., La Rosa F., Santangelo M.A., Caputo D., Mendozzi L., Rovaris M., Clerici M. (2018). Monosodium Urate Crystals Activate the Inflammasome in Primary Progressive Multiple Sclerosis. Front. Immunol..

[34] Murakami T., Ruengsinpinya L., Nakamura E., Takahata Y., Hata K., Okae H., Taniguchi S., Takahashi M., Nishimura R. (2019). Cutting Edge: G Protein Subunit β 1 Negatively Regulates NLRP3 Inflammasome Activation. J. Immunol..

[35] Jhang J.-J., Cheng Y.-T., Ho C.-Y., Yen G.-C. (2015). Monosodium urate crystals trigger Nrf2- and heme oxygenase-1-dependent inflammation in THP-1 cells. Cell. Mol. Immunol..

[36] Feng S., Zhang Z., Mo Y., Tong R., Zhong Z., Chen Z., He D., Wan R., Gao M., Mo Y. (2020). Activation of NLRP3 inflammasome in hepatocytes after exposure to cobalt nanoparticles: The role of oxidative stress. Toxicol. Vitro.

[37] Klasson M., Lindberg M., Westberg H., Bryngelsson I.L., Tuerxun K., Persson A., Särndahl E. (2021). Dermal exposure to cobalt studied in vitro in keratinocytes-effects of cobalt exposure on inflammasome activated cytokines, and mRNA response. Biomarkers.

[38] Cappellini F., Hedberg Y., McCarrick S., Hedberg J., Derr R., Hendriks G., Odnevall Wallinder I., Karlsson H.L. (2018). Mechanistic insight into reactivity and (geno)toxicity of well-characterized nanoparticles of cobalt metal and oxides. Nanotoxicology.

[39] Francis W.R., Liu Z., Owens S.E., Wang X., Xue H., Lord A., Kanamarlapudi V., Xia Z. (2021). Role of hypoxia inducible factor 1α in cobalt nanoparticle induced cytotoxicity of human THP-1 macrophages. Biomater. Transl..

[40] Karri V., Lidén C., Fyhrquist N., Högberg J., Karlsson H.L. (2021). Impact of mono-culture vs. Co-culture of keratinocytes and monocytes on cytokine responses induced by important skin sensitizers. J. Immunot..

[41] Samelko L., Caicedo M.S., Lim S.-J., Della-Valle C., Jacobs J., Hallab N.J. (2013). Cobalt-Alloy Implant Debris Induce HIF-1α Hypoxia Associated Responses: A Mechanism for Metal-Specific Orthopedic Implant Failure. PLoS One.

[42] Swanson K.V., Deng M., Ting J.P.Y. (2019). The NLRP3 inflammasome: molecular activation and regulation to therapeutics. Nat. Rev. Immunol..

[43] Puren A.J., Fantuzzi G., Dinarello C.A. (1999). Gene expression, synthesis, and secretion of interleukin 18 and interleukin 1beta are differentially regulated in human blood mononuclear cells and mouse spleen cells. Proc. Natl. Acad. Sci. USA.

[44] Martinon F., Burns K., Tschopp J. (2002). The inflammasome: a molecular platform triggering activation of inflammatory caspases and processing of proIL-beta. Mol. Cell.

[45] Gritsenko A., Yu S., Martin-Sanchez F., Diaz-del-Olmo I., Nichols E.-M., Davis D.M., Brough D., Lopez-Castejon G. (2020). Priming Is Dispensable for NLRP3 Inflammasome Activation in Human Monocytes In Vitro. Front. Immunol..

[46] Seal S., Carreras-Puigvert J., Trapotsi M.-A., Yang H., Spjuth O., Bender A. (2022). Integrating cell morphology with gene expression and chemical structure to aid mitochondrial toxicity detection. Commun. Biol..

[47] Mishra S.R., Mahapatra K.K., Behera B.P., Patra S., Bhol C.S., Panigrahi D.P., Praharaj P.P., Singh A., Patil S., Dhiman R., Bhutia S.K. (2021). Mitochondrial dysfunction as a driver of NLRP3 inflammasome activation and its modulation through mitophagy for potential therapeutics. Int. J. Biochem. Cell Biol..

[48] Jiang H., Song D., Zhou X., Chen F., Yu Q., Ren L., Dai Q., Zeng M. (2023). Maresin1 ameliorates MSU crystal-induced inflammation by upregulating Prdx5 expression. Mol. Med..

[49] Ponti J., Sabbioni E., Munaro B., Broggi F., Marmorato P., Franchini F., Colognato R., Rossi F. (2009). Genotoxicity and morphological transformation induced by cobalt nanoparticles and cobalt chloride: an in vitro study in Balb/3T3 mouse fibroblasts. Mutagenesis.

[50] Coll R.C., Robertson A., Butler M., Cooper M., O'Neill L.A.J. (2011). The Cytokine Release Inhibitory Drug CRID3 Targets ASC Oligomerisation in the NLRP3 and AIM2 Inflammasomes. PLoS One.

[51] Tapia-Abellán A., Angosto-Bazarra D., Martínez-Banaclocha H., de Torre-Minguela C., Cerón-Carrasco J.P., Pérez-Sánchez H., Arostegui J.I., Pelegrin P. (2019). MCC950 closes the active conformation of NLRP3 to an inactive state. Nat. Chem. Biol..

[52] Coll R.C., Hill J.R., Day C.J., Zamoshnikova A., Boucher D., Massey N.L., Chitty J.L., Fraser J.A., Jennings M.P., Robertson A.A.B., Schroder K. (2019). MCC950 directly targets the NLRP3 ATP-hydrolysis motif for inflammasome inhibition. Nat. Chem. Biol..

[53] Caicedo M.S., Desai R., McAllister K., Reddy A., Jacobs J.J., Hallab N.J. (2009). Soluble and particulate Co-Cr-Mo alloy implant metals activate the inflammasome danger signaling pathway in human macrophages: a novel mechanism for implant debris reactivity. J. Orthop. Res..

[54] Kim E.H., Won J.H., Hwang I., Yu J.W. (2013). Cobalt Chloride-induced Hypoxia Ameliorates NLRP3-Mediated Caspase-1 Activation in Mixed Glial Cultures. Immune Netw..

[55] Seal S., Carreras-Puigvert J., Singh S., Carpenter A.E., Spjuth O., Bender A. (2024). From pixels to phenotypes: Integrating image-based profiling with cell health data as BioMorph features improves interpretability. Mol. Biol. Cell.

[56] Menu P., Mayor A., Zhou R., Tardivel A., Ichijo H., Mori K., Tschopp J. (2012). ER stress activates the NLRP3 inflammasome via an UPR-independent pathway. Cell Death Dis..

[57] Burger D., Fickentscher C., de Moerloose P., Brandt K.J. (2016). F-actin dampens NLRP3 inflammasome activity via Flightless-I and LRRFIP2. Sci. Rep..

[58] Herring M., Persson A., Potter R., Karlsson R., Särndahl E., Ejdebäck M. (2024). Exposing kinetic disparities between inflammasome readouts using time-resolved analysis. Heliyon.

[59] Xia J., Psychogios N., Young N., Wishart D.S. (2009). MetaboAnalyst: a web server for metabolomic data analysis and interpretation. Nucleic Acids Res..

[60] Stirling D.R., Swain-Bowden M.J., Lucas A.M., Carpenter A.E., Cimini B.A., Goodman A. (2021). CellProfiler 4: improvements in speed, utility and usability. BMC Bioinf..

[61] Alijagic A., Kotlyar O., Larsson M., Salihovic S., Hedbrant A., Eriksson U., Karlsson P., Persson A., Scherbak N., Färnlund K. (2024). Immunotoxic, genotoxic, and endocrine disrupting impacts of polyamide microplastic particles and chemicals. Environ. Int..

[62] Behdenna A., Colange M., Haziza J., Gema A., Appé G., Azencott C.A., Nordor A. (2023). pyComBat, a Python tool for batch effects correction in high-throughput molecular data using empirical Bayes methods. BMC Bioinf..

[63] McInnes L., Healy J., Saul N., Großberger L. (2020). UMAP: Uniform Manifold Approximation and Projection for Dimension Reduction. arXiv.

