# Supplementary material for: Exploring NLRP3-related phenotypic fingerprints in human macrophages using Cell Painting assay
Source: iScience. 2025 Feb 5;28(3):111961. doi: 10.1016/j.isci.2025.111961 (PMC11876907; doi:10.1016/j.isci.2025.111961)
Supplement: Document S1. Figures S1 and S2 [file mmc1.pdf]

**iScience, Volume *28***

## Supplemental information

# Exploring NLRP3-related phenotypic fingerprints in human macrophages using Cell Painting assay

**Matthew Herring, Eva Särndahl, Oleksandr Kotlyar, Nikolai Scherbak, Magnus Engwall, Roger Karlsson, Mikael Ejdebäck, Alexander Persson, and Andi Alijagic**

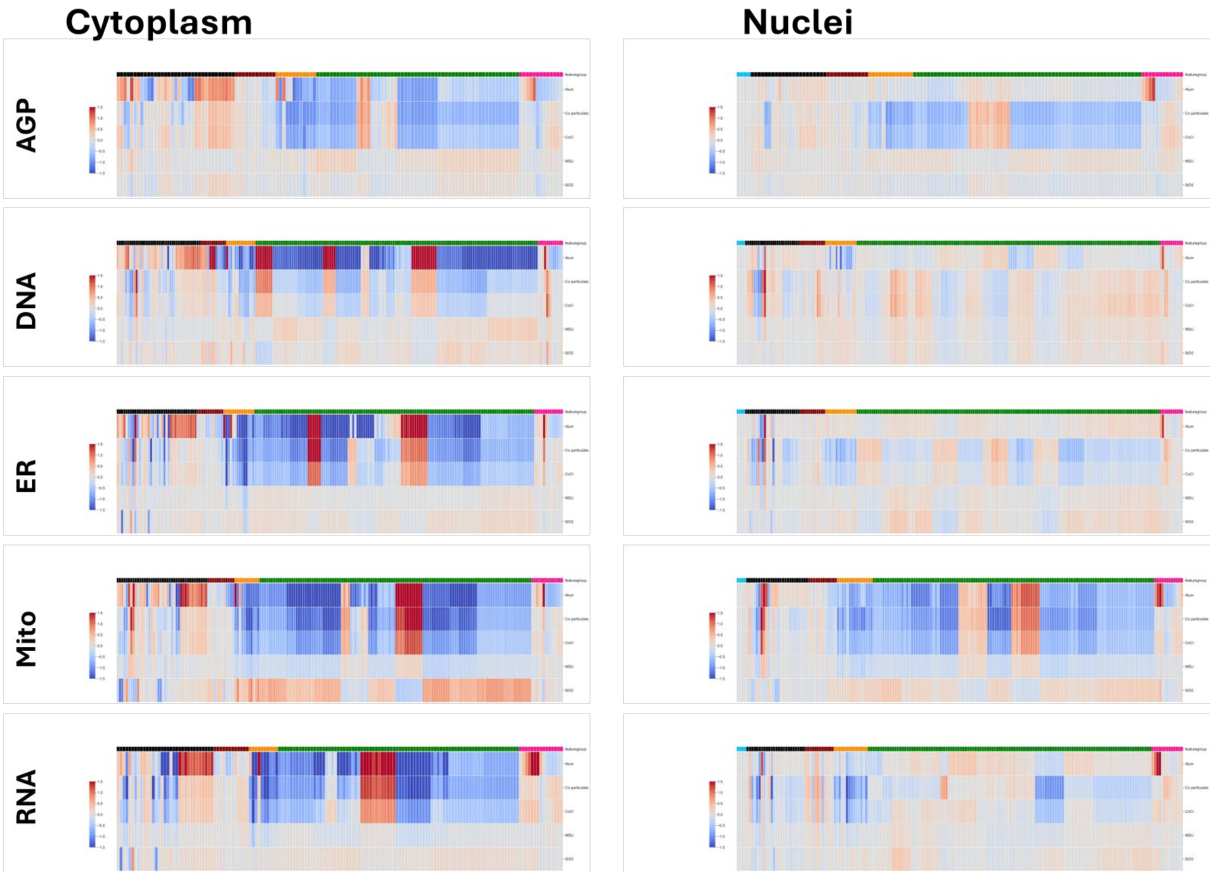


**Supplementary figure S1, related to Figure 3.** The heatmaps were constructed with phenotypic features, retrieved from Cell Painting single-cell profiles of THP-1 macrophages exposed to various triggers. The features represent the “Cytoplasm” and “Nuclei” compartments of features extracted using CellProfiler. The feature values are shown at the treatment-level aggregation. The features were grouped into five channels: AGP, DNA, RNA, Mito, and ER. Each color on the heatmap indicates the fold change, either an increase (red) or decrease (blue), of the respective phenotypic feature compared to the unexposed control. Each column in the heatmap corresponds to a specific phenotypic feature.

# Features altered by NLRP3 triggers

| **Trigger:** Alum (particulate) |
|---|
| **Number of altered features (p<0.05)** |
| 1433 of 2584 |
| **Top 10 altered features** |
| *Cytoplasm_RadialDistribution_FracAtD_mito_tubeness_14of16* |
| *Cytoplasm_RadialDistribution_FracAtD_mito_tubeness_18of20* |
| *Cytoplasm_RadialDistribution_FracAtD_mito_tubeness_17of20* |
| *Cells_RadialDistribution_FracAtD_mito_tubeness_11of16* |
| *Cells_Texture_InfoMeas1_Mito_5_02_256* |
| *Cells_Intensity_MedianIntensity_RNA* |
| *Cells_Texture_InfoMeas1_Mito_3_03_256* |
| *Cytoplasm_Texture_InfoMeas2_DNA_10_01_256* |
| *Cytoplasm_AreaShape_Zernike_4_0* |
| *Cells_AreaShape_Zernike_4_4* |

| **Trigger:** Co particles (particulate) |
|---|
| **Number of altered features (p<0.05)** |
| 1483 of 2584 |
| **Top 10 altered features** |
| *Cells_Intensity_MedianIntensity_Mito* |
| *Cells_Intensity_MADIntensity_Mito* |
| *Cytoplasm_AreaShape_Zernike_4_4* |
| *Cytoplasm_Intensity_LowerQuartileIntensity_RNA* |
| *Cells_AreaShape_Zernike_4_4* |
| *Cells_AreaShape_Zernike_6_6* |
| *Cells_Intensity_LowerQuartileIntensity_RNA* |
| *Cells_Texture_InfoMeas1_Mito_3_02_256* |
| *Cells_Correlation_RWC_RNA_ER* |
| *Cells_Texture_InfoMeas1_Mito_3_00_256* |

| **Trigger:** $CoCl_2$ (chemical) |
|---|
| **Number of altered features (p<0.05)** |
| 677 of 2584 |
| **Top 10 altered features** |
| *Cytoplasm_RadialDistribution_FracAtD_mito_tubeness_14of16* |
| *Cells_Intensity_LowerQuartileIntensity_ER* |
| *Cytoplasm_Intensity_LowerQuartileIntensity_ER* |
| *Nuclei_Texture_Entropy_Mito_5_03_256* |
| *Nuclei_Texture_Entropy_Mito_5_01_256* |
| *Nuclei_Texture_Entropy_Mito_5_00_256* |
| *Cells_Intensity_LowerQuartileIntensity_DNA* |
| *Nuclei_Texture_Entropy_Mito_3_01_256* |
| *Nuclei_Texture_Entropy_Mito_5_02_256* |
| *Nuclei_Texture_Entropy_Mito_3_03_256* |

| **Trigger:** $SiO_2$ (particulate) |
|---|
| **Number of altered features (p<0.05)** |
| 547 of 2584 |
| **Top 10 altered features** |
| *Cells_RadialDistribution_MeanFrac_mito_tubeness_9of16* |
| *Cells_RadialDistribution_MeanFrac_mito_tubeness_11of20* |
| *Cells_RadialDistribution_MeanFrac_mito_tubeness_12of20* |
| *Cells_Granularity_6_DNA* |
| *Nuclei_Granularity_6_DNA* |
| *Cells_RadialDistribution_MeanFrac_mito_tubeness_10of16* |
| *Cells_RadialDistribution_FracAtD_mito_tubeness_9of16* |
| *Cells_RadialDistribution_FracAtD_mito_tubeness_11of20* |
| *Cells_RadialDistribution_MeanFrac_DNA_1of4* |
| *Cells_RadialDistribution_MeanFrac_mito_tubeness_8of20* |

| **Trigger:** MSU (particulate; crystal-like) |
|---|
| **Number of altered features (p<0.05)** |
| 61 of 2584 |
| **Top 10 altered features** |
| *Cytoplasm_RadialDistribution_FracAtD_mito_tubeness_17of20* |
| *Cells_RadialDistribution_MeanFrac_mito_tubeness_12of20* |
| *Cells_RadialDistribution_FracAtD_mito_tubeness_9of16* |
| *Cells_RadialDistribution_MeanFrac_DNA_2of4* |
| *Cells_RadialDistribution_FracAtD_mito_tubeness_12of20* |
| *Cells_RadialDistribution_MeanFrac_mito_tubeness_11of20* |
| *Cells_RadialDistribution_FracAtD_mito_tubeness_11of20* |
| *Cells_RadialDistribution_MeanFrac_mito_tubeness_9of16* |
| *Cytoplasm_AreaShape_Zernike_4_4* |
| *Cells_RadialDistribution_MeanFrac_mito_tubeness_10of16* |

**Supplementary figure S2, related to Figure 4.** Features altered by NLRP3-triggers. The top ten altered features in THP-1 macrophages after exposure to the NLRP3 triggers Alum, cobalt (Co) particulate, $CoCl_2$, $SiO_2$ and MSU.

# Features altered by NLRP3-triggers after LPS-priming

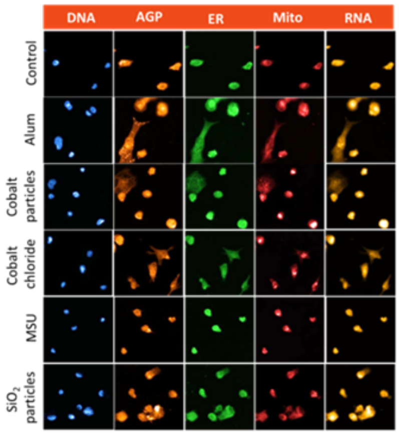


| **Trigger:** Alum (particulate) |
|---|
| **Number of altered features (p<0.05)** |
| 1952 of 2584 |
| **Top 10 altered features** |
| *Cytoplasm_RadialDistribution_MeanFrac_DNA_2of4* |
| *Cytoplasm_RadialDistribution_MeanFrac_DNA_1of4* |
| *Cytoplasm_Texture_Correlation_RNA_3_00_256* |
| *Cytoplasm_Texture_Correlation_RNA_5_00_256* |
| *Cytoplasm_Texture_InverseDifferenceMoment_DNA_3_00_256* |
| *Cytoplasm_Texture_Correlation_RNA_3_02_256* |
| *Cytoplasm_Texture_InverseDifferenceMoment_DNA_3_01_256* |
| *Cytoplasm_Texture_Correlation_RNA_3_01_256* |
| *Cytoplasm_Texture_InverseDifferenceMoment_DNA_3_03_256* |
| *Cytoplasm_Texture_Correlation_RNA_5_02_256* |

| **Trigger:** Co particles (particulate) |
|---|
| **Number of altered features (p<0.05)** |
| 1838 of 2584 |
| **Top 10 altered features** |
| *Cytoplasm_Texture_InverseDifferenceMoment_Mito_3_02_256* |
| *Cytoplasm_Texture_InverseDifferenceMoment_DNA_5_01_256* |
| *Cytoplasm_Texture_InverseDifferenceMoment_Mito_3_03_256* |
| *Cytoplasm_Texture_InverseDifferenceMoment_DNA_5_02_256* |
| *Cytoplasm_Texture_InverseDifferenceMoment_DNA_5_03_256* |
| *Cytoplasm_Texture_InfoMeas1_RNA_5_03_256* |
| *Cytoplasm_Texture_Correlation_Mito_5_00_256* |
| *Cytoplasm_Texture_InverseDifferenceMoment_Mito_3_00_256* |
| *Cytoplasm_Texture_InverseDifferenceMoment_Mito_3_01_256* |
| *Cytoplasm_Texture_InverseDifferenceMoment_DNA_5_00_256* |

| **Trigger:** $CoCl_2$ (chemical) |
|---|
| **Number of altered features (p<0.05)** |
| 1668 of 2584 |
| **Top 10 altered features** |
| *Cells_Texture_Correlation_Mito_5_02_256* |
| *Nuclei_RadialDistribution_RadialCV_DNA_1of4* |
| *Cytoplasm_Texture_Correlation_Mito_5_03_256* |
| *Cells_Texture_Correlation_Mito_3_01_256* |
| *Cytoplasm_Texture_Correlation_Mito_5_01_256* |
| *Cytoplasm_Texture_Correlation_RNA_10_02_256* |
| *Cytoplasm_Texture_InfoMeas1_RNA_3_00_256* |
| *Cytoplasm_Texture_InfoMeas1_RNA_3_01_256* |
| *Cytoplasm_Texture_InfoMeas1_RNA_3_02_256* |
| *Cells_Texture_InfoMeas1_RNA_3_02_256* |

| **Trigger:** $SiO_2$ (particulate) |
|---|
| **Number of altered features (p<0.05)** |
| 24 of 2584 |
| **Top 10 altered features** |
| *Nuclei_RadialDistribution_RadialCV_DNA_1of4* |
| *Cytoplasm_RadialDistribution_FracAtD_mito_tubeness_14of20* |
| *Nuclei_RadialDistribution_RadialCV_DNA_2of4* |
| *Cytoplasm_Texture_Correlation_Mito_10_02_256* |
| *Cytoplasm_Texture_Correlation_RNA_5_03_256* |
| *Cytoplasm_Texture_Correlation_RNA_5_01_256* |
| *Cytoplasm_Texture_Correlation_Mito_10_00_256* |
| *Cytoplasm_Intensity_IntegratedIntensityEdge_Mito* |
| *Nuclei_Granularity_2_DNA* |
| *Cytoplasm_Texture_Correlation_RNA_10_00_256* |

| **Trigger:** MSU (particulate; crystal-like) |
|---|
| **Number of altered features (p<0.05)** |
| 120 of 2584 |
| **Top 10 altered features** |
| *Nuclei_Texture_Correlation_DNA_10_03_256* |
| *Cells_RadialDistribution_MeanFrac_mito_tubeness_17of20* |
| *Nuclei_Texture_Correlation_DNA_10_01_256* |
| *Cytoplasm_Intensity_MassDisplacement_Mito* |
| *Cells_RadialDistribution_MeanFrac_mito_tubeness_14of16* |
| *Cells_AreaShape_Zernike_7_1* |
| *Cells_RadialDistribution_FracAtD_mito_tubeness_14of16* |
| *Cytoplasm_Texture_Correlation_Mito_5_02_256* |
| *Cytoplasm_AreaShape_Zernike_8_6* |
| *Cells_RadialDistribution_FracAtD_mito_tubeness_17of20* |

**Supplementary figure S3, related to Figure 5**. Features altered by NLRP3-triggers after LPS-priming. Representative images of THP-1 macrophages, as well as the top ten altered features after LPS-priming and exposure to the NLRP3 triggers Alum, cobalt (Co) particles, $CoCl_2$, $SiO_2$ and MSU.

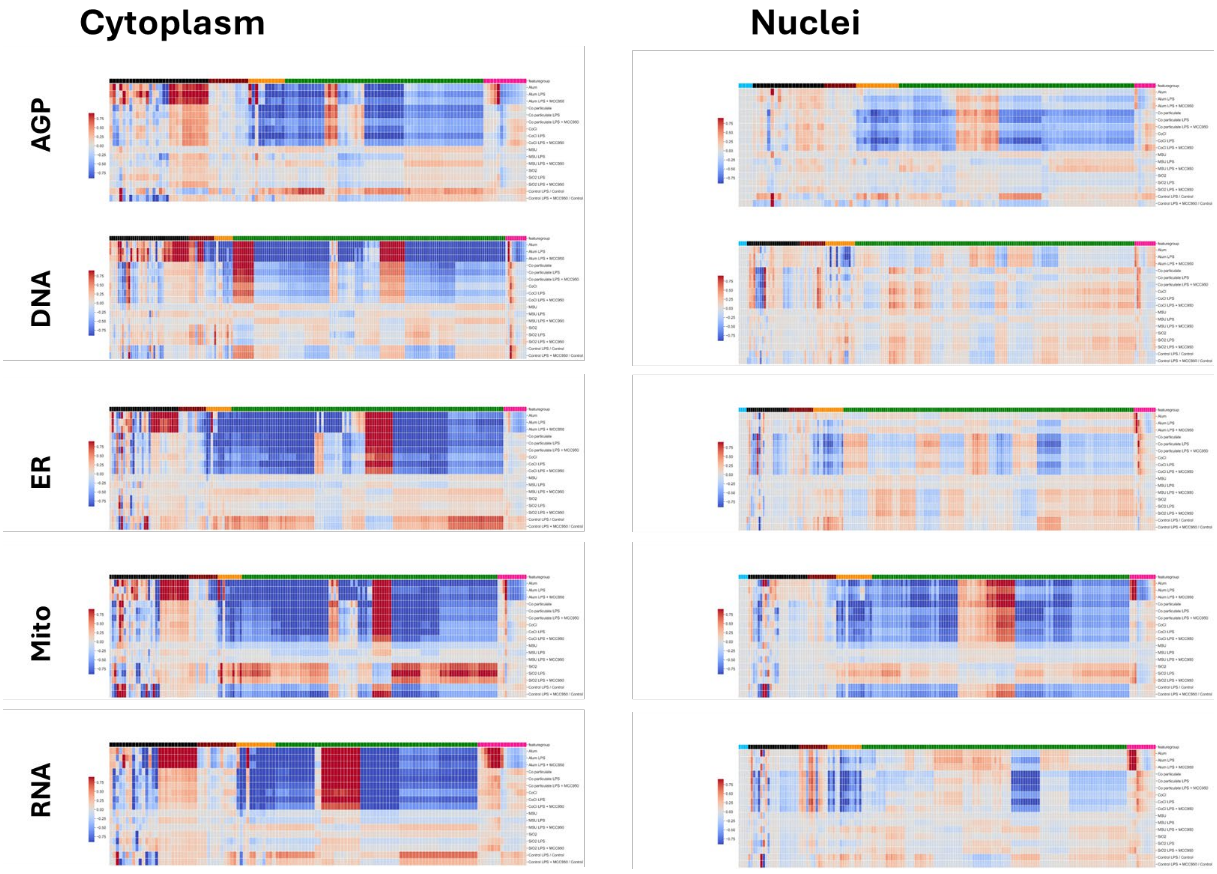


**Supplementary figure S4, related to Figure 5.** The heatmaps were constructed with phenotypic features, retrieved from Cell Painting single-cell profiles of THP-1 macrophages exposed to various triggers. The features represent the “Cytoplasm” and “Nuclei” compartments of features extracted using CellProfiler. The feature values are shown at the treatment-level aggregation. The features were grouped into five channels: AGP, DNA, RNA, Mito, and ER. Each color on the heatmap indicates the fold change, either an increase (red) or decrease (blue), of the respective phenotypic feature compared to the unexposed control. Each column in the heatmap corresponds to a specific phenotypic feature.

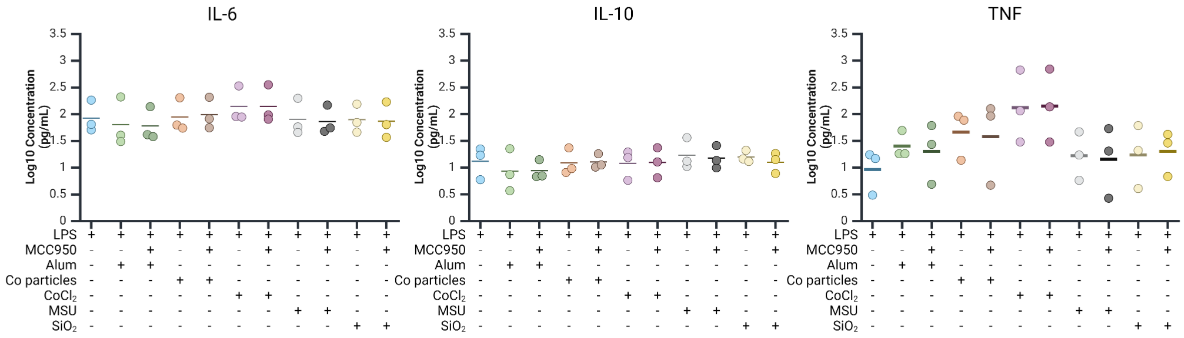


**Supplementary Figure S5. Cytokine expression, related to Figure 7.** Levels of extracellular IL-6, IL-10 and TNF following either lipopolysaccharide (LPS) priming alone, LPS priming followed by exposure to Alum, cobalt (Co) particles, $CoCl_2$, MSU, or $SiO_2$, or LPS priming and MCC950 inhibition, followed by

exposure to Alum, cobalt particles, $CoCl_2$, MSU, or $SiO_2$. Significance for LPS is compared to control, while significance for the remaining treatments without MCC950 are compared to LPS. Significance for treatments with MCC950 are compared to the equivalent trigger, with LPS but without MCC950. Comparisons between treatments without MCC950 and LPS were analyzed by one-way ANOVA with Dunnett multiple comparisons test. Remaining data was analyzed by t-test followed by Holm-Šídák multiple comparisons test. Figure was created with BioRender.com
